# Changing the thickness of two layers: i-ZnO nanorods, p-Cu_2_O and its influence on the carriers transport mechanism of the p-Cu_2_O/i-ZnO nanorods/n-IGZO heterojunction

**DOI:** 10.1186/s40064-016-2468-y

**Published:** 2016-06-13

**Authors:** Nguyen Huu Ke, Le Thi Tuyet Trinh, Pham Kim Phung, Phan Thi Kieu Loan, Dao Anh Tuan, Nguyen Huu Truong, Cao Vinh Tran, Le Vu Tuan Hung

**Affiliations:** Department of Applied Physics, Faculty of Physics and Engineering Physics, University of Science, VNU-HCM, 227 Nguyen Van Cu Street, Award 4, District 5, Ho Chi Minh City, Viet Nam; Laboratory of Advanced Materials, University of Science, VNU-HCM, 227 Nguyen Van Cu Street, Award 4, District 5, Ho Chi Minh City, Viet Nam

**Keywords:** Electrochemical method, ZnO nanorods, Heterojunction, Cu_2_O layer, Solar cells

## Abstract

In this study, two layers: i-ZnO nanorods and p-Cu_2_O were fabricated by electrochemical deposition. The fabricating process was the initial formation of ZnO nanorods layer on the n-IGZO thin film which was prepared by sputtering method, then a p-Cu_2_O layer was deposited on top of rods to form the p-Cu_2_O/i-ZnO nanorods/n-ZnO heterojunction. The XRD, SEM, UV–VIS, I–V characteristics methods were used to define structure, optical and electrical properties of these heterojunction layers. The fabricating conditions and thickness of the Cu_2_O layers significantly affected to the formation, microstructure, electrical and optical properties of the junction. The length of i-ZnO nanorods layer in the structure of the heterojunction has strongly affected to the carriers transport mechanism and performance of this heterojunction.

## Background

Renewable energy is expected to replace depleting fossil energy sources in order to ensure energy security and overcome the problem of global climate change. Currently, when the demand for energy is increasing, the manufacture of cheap and durable solar cells is an essential requirement. As opposed to the high cost of single crystal silicon, the metal oxide semiconductors are suitable options for solar cells fabrication because of the diversity and simplicity in manufacturing of them (Abdu and Musa 2009). The metal oxide semiconductors prepared by thin film technology can save material and production costs. In addition, the structure of these semiconductors can be easily adjusted. Therefore, the suitable electrical and optical properties are easily obtained for forming the optoelectronic devices based on heterojunctions (Chen 2013).

Among the oxide semiconductors, zinc oxide (ZnO) and cuprous oxide (Cu_2_O) are attractive of many scientists because they have favorable photoelectric properties and economic values, such as suitable bandgap, good thermal stability, low-cost and environment-friendly material (Wang et al. 2011). ZnO is an n-type semiconductor with a direct bandgap. The bandgap energy of ZnO is about 3.37 eV corresponding to exciton bounding energy of 60 meV. The improving in optical and electronic properties of ZnO by doping metal atoms such as Ga, In, Al etc. made it specially suitable for n-type electrode materials of solar cells because of the high transmittance in the visible wavelength region and high electron concentration (Kidowaki et al. 2012). Especially, the 1D ZnO nanostructures that have the larger surface area and high electron mobility are promising in enhancing the ability to the separation and transmission of carriers (Baek et al. 2013). However, there are many difficult problems in preparing of the p-type ZnO semiconductor that lead to unstable electrical capacity. Therefore, it is difficult to get homojunction based on n-type and p-type of ZnO (Gershon et al. 2013). As noted above, the p-type ZnO layer need to be replaced by another semiconductor. Among metal oxide semiconductors, Cu_2_O shows up as a bright candidate. Naturally, Cu_2_O is a p-type semiconductor due to the present of Cu^+^ vacancy in crystalline structure. Its potential for solar cells was revived during the mid-seventies as a possible low-cost material (De Jongh et al. 1999). The bandgap of Cu_2_O semiconductor is about 2.17 eV and this kind of semiconductor has absorption edge in visible range. The absorption coefficient of Cu_2_O is higher than single crystalline Si therefore it has been considered as a potential material for the light absorbing layer in solar cells (Zoolfakar et al. 2012).

Base on Shockley–Queisser theory, the power conversion efficiency is about 20 % could be obtained from the thin film solar cell made of n-type ZnO and p-type Cu_2_O heterojunction (Cheng et al. 2013). In such heterojunction cells, the Cu_2_O layers are generally prepared by many physical and chemical techniques such as thermal oxidation of metallic Cu sheet, DC and RF sputtering, pulse laser deposition, photochemical deposition, chemical vapor deposition, and electrochemical deposition. Among them, the electrochemical deposition method has several advantages such as low cost, saving material, simple fabrication, and easy application (Jeong et al. 2008). However, results from many reports have showed that the conversion efficiency of ZnO/Cu_2_O heterojunction prepared by electrochemical method was still low in range 0.007–0.2 % because of two main reasons: the quality of crystal structure affected the electrical conductivity and absorption capacity of the Cu_2_O layer, and the defects at interface between two layers trapped carriers and produced a tunnel recombination process (Lv et al. 2013).

In present work, a ZnO nanorods layer was deposited in the middle of two layers: n-IGZO and p-Cu_2_O to investigate the carriers transport mechanisms and performance of the junction. The ZnO nanorods layer was also prepared by electrochemical deposition. This layer worked as intrinsic layer and had effective contributions in separate, transport mechanisms of carriers.

## Methods

**Fig. 1:**
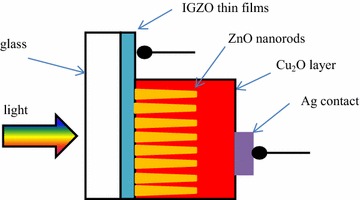
Schematic diagram of the p-Cu_2_O/i-ZnO nanorods/n-IGZO heterojunction

First, Indium–Gallium–Zinc Oxide (IGZO) thin films with sheet resistance of 10 Ω/square and Pt foil were used as a working electrode and a counter electrode, respectively. IGZO thin films, sputtered from the ceramic targets, were deposited on glass substrates (Marienfeld, Germany) using DC magnetron sputtering. The films were deposited in pure Ar gas plasma with a sputtering pressure of 0.4 Pa and power density of 1.32 W/cm^2^. The substrate temperature 300 °C and the target-substrate distance 5 cm are constant during deposition (Pham et al. 2014). The electrical properties IGZO thin films, including carrier concentration, mobility, and resistivity are corresponding to 8 × 10^20^ cm^−3^, 25 cm^2^/V s and 7.5 × 10^−4^ Ω cm (Fig. 1).

Second, the i-ZnO nanorods layer was grown on IGZO substrates by using the electrochemical deposition. The electrolyte was prepared by adding of 0.05 M Zn(NO_3_)_2_·6H_2_O and 0.05 M C_6_H_12_N_4_. This solution in electrolytic tank was heated to a temperature of 80 °C. In the solution, there were many happened processes, first Zn(NO_3_)_2_ was dissociated to form two Zn^2+^ and NO_3_^−^ ions then NO_3_^−^ ions combined with water in solution and two electrons to form two ions: NO_2_^−^ and OH^−^. Besides, C_6_H_12_N_4_ decomposed into NH_3_ and HCHO. The NH_3_ reacted with water to produce NH^4+^ and OH^−^. Through two process, OH^−^ ions were continuously provided for the formation of Zn(OH)_2_ which subsequently formed ZnO. The ZnO nanorods layer was electrodeposited at 80 °C for 60 min. After that, the sample was rinsed with distilled water and transferred in the Cu_2_O electrodeposition bath. The Cu_2_O layer was prepared in solution of Copper (II) sulfate (CuSO_4_, 0.02 mol L^−1^) and Lactic acid (4 mol L^−1^). The pH of the electrochemical solution was adjusted to 11 by adding NaOH. The electrolyte temperature was kept at 70 °C during electrochemical process. The current density at 0.1 and 0.15 mA cm^−2^ for two steps were set up to growth of Cu_2_O crystals. In step 1, the seed layer was prepared according to current density of 0.1 mA cm^−2^ in order to make the bonding ability between Cu_2_O seeds and ZnO nanorods. After that, the thickness of Cu_2_O was grown by step 2 and the sample thickness could be adjusted by the change of deposition time (Jeong et al. 2013). The silver paste was used as a back contact of the p-Cu_2_O/i-ZnO nanorods/n-IGZO heterojunction.

The morphology and size of the product were analyzed by using scanning electron microscopy (SEM). X-ray diffraction (XRD) patterns to determine the crystalline structure of the samples were obtained by using a D8 ADVANCE-BRUKER system with Cu Kα primary X-rays. The optical spectra were recorded by using UV–Vis Jasco V-530 in the wavelength range of 200 to 1100 nm. The Keithley K2612A source and Agilent 4294 Precision Impedance Analyser were used to measure the electrical properties of the heterojunction. The photoelectric properties were performed by using a solar simulator (XES-40S1, San-Ei) equipped with AM 1.5 G filters used at 100 mW/cm^2^. The solar cells were illuminated through the side of the IGZO substrate, and the illuminated area was 0.25 cm^2^.

## Results and discussion

### Morphology and crystal structure

**Fig. 2:**
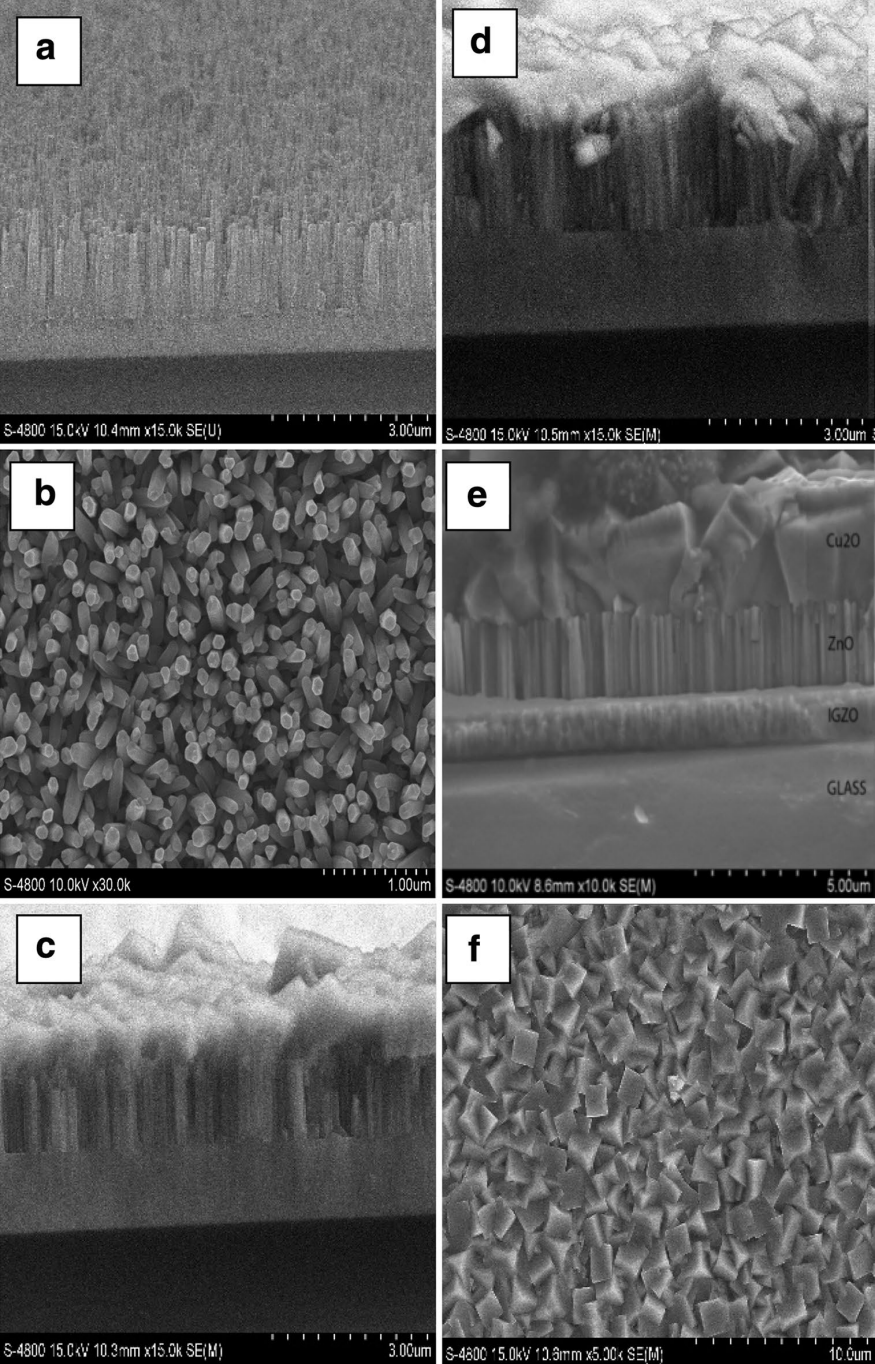
SEM images of the p-Cu_2_O/i-ZnO nanorods/n-IGZO heterojunction. **a** ZnO nanorods on IGZO substrate. **b** Top-view of ZnO nanorods. **c** The cross-section image of the p-Cu_2_O/i-ZnO nanorods/n-IGZO with growth-time of ZnO nanorods is 2000 s. **d** The cross-section image of the p-Cu_2_O/i-ZnO nanorods/n-IGZO with growth-time of ZnO nanorods is 4000 s. **e** The cross-section image of the p-Cu_2_O/i-ZnO nanorods/n-IGZO with growth-time of Cu_2_O is increased to 6 h. **f** Top-view of Cu_2_O surface

The SEM images of three distinct layers in the p-Cu_2_O/i-ZnO nanorods/n-IGZO heterojunction were shown in Fig. 2. The ZnO nanorods were growth on IGZO substrate with vertical direction. The single nanorod is about 1.5 μm in length. The top of nanorods has a hexagonal morphology with a diameter of approximately 100 nm. The rod-to-rod space is around 300–500 nm according to Fig. 2b. An absorber layer of Cu_2_O was evenly deposited on the surface of the ZnO nanorods by electrochemical method. Figure 2c, d have shown that the Cu_2_O layer was full fill into space of rods. The length of ZnO nanorods was changed in range 1–2 μm when depositing time of these rods was increased from 2000 to 4000 s. The thickness of Cu_2_O layer was also improved to 5 μm clearly when the growth-time of Cu_2_O layer in the bath was adjusted from 2 to 6 h as Fig. 2e.

The top-view of Cu_2_O surface revealed the fact that the cubic crystalline structure of Cu_2_O was obtained from electrochemical method. The size of Cu_2_O crystal is about 1–2 μm and no ZnO nanorod is observed on the surface have proved the well-contact between two layers: ZnO nanorods and Cu_2_O. In confirmation, uniformly distinctive hexagonal morphologies with clear boundaries observed in the SEM images will lead to decreasing of defects at interface and improving charge collection efficiency of heterojunction (Baek et al. 2013).

**Fig. 3:**
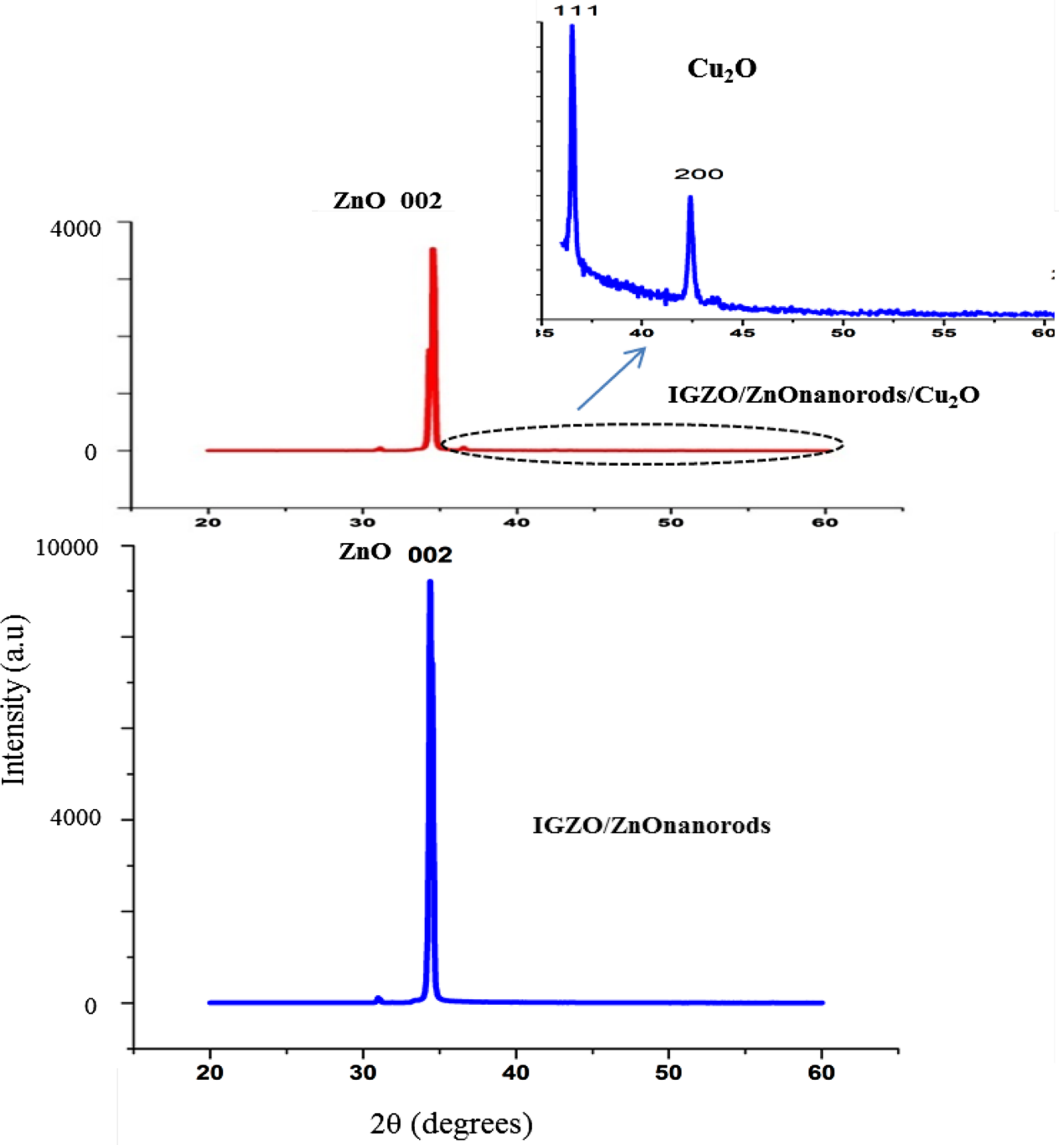
XRD patterns of the ZnO nanorods on IGZO glass and the p-Cu_2_O/i-ZnO nanorods/n-IGZO heterojunction

Figure 3 has shown the XRD patterns of both structures: the ZnO nanorods on IGZO substrate and the Cu_2_O/ZnO nanorods/IGZO.

In the case of the ZnO nanorods on IGZO substrate, the high intensity peak at 2θ = 34.3^o^ revealed the fact that ZnO nanorods have wurtzite structure and orientation of (002) plane. When a Cu_2_O layer was deposited on top of ZnO nanorods, the intensity of ZnO (002) peak was also decreased clearly due to the Cu_2_O layer on the ZnO nanorods. The effect of IGZO substrate and ZnO nanorods layer was so strong that no peak of Cu_2_O structure was observed clearly. However, the XRD pattern of Cu_2_O/ZnO nanorods/IGZO heterojunction in range of 2θ from 35° to 60° has revealed cubic structure of Cu_2_O at (111) and (200) planes according to 2θ = 36.45° and 42.35° (Jeong et al. 2013). The weak peak at Cu_2_O (111) orientation compared with the strong ZnO (002) peak has indicated that the seeds layer of Cu_2_O was formed on the surface of ZnO nanorods and small crystals of Cu_2_O were randomly distributed on interface of heterojunction (Perng et al. 2013). This lead to some advances in carriers transport capability of solar cell based on the p-Cu_2_O/i-ZnO nanorods/n-IGZO heterojunction.

### Optical properties

**Fig. 4:**
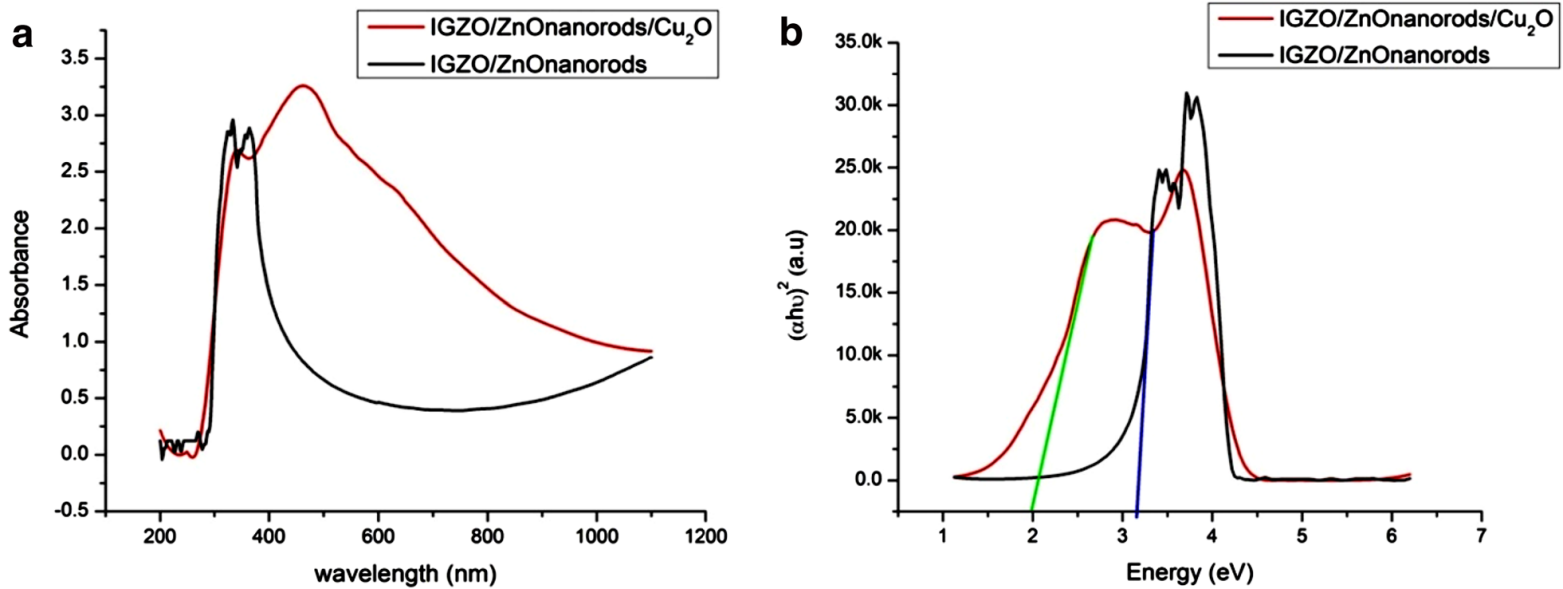
**a** Absorption spectra and **b** Tauc plots of the ZnO nanorods on IGZO glass and the p-Cu2O/i-ZnO nanorods/n-IGZO heterojunction

The absorption spectra of two structures: IGZO/ZnOnanorods and IGZO/ZnO nanorods/Cu_2_O are presented in Fig. 4a. The optical absorption peak at 370 nm is due to contribution of ZnO crystal phase including IGZO substrate and ZnO nanorods, and a peak at 470–500 nm corresponds to the Cu_2_O absorption layer (Noda et al. 2013). Clearly, when the Cu_2_O was deposited on the top of ZnO nanorods layer, the absorption peak was enhanced obviously. Especially, the absorption range of the IGZO/ZnO nanorods/Cu_2_O heterojunction was expanded from 400 to 800 nm because of the high absorption coefficient in the visible range of the Cu_2_O layer (Oku et al. 2014). For determination of the bandgap energy (Eg) of ZnO and Cu_2_O in the heterostructure, the method based on the relation of αhυ = A(hυ − Eg)^n/2^ was used. Where n is a number that depends on the nature of the transition (Noda et al. 2013). In this case, the value of n was found to be 1 because of the direct band to band transition happening in ZnO and Cu_2_O semiconductors. Figure 4b is a Tauc plot, which shows (αhν)^2^ versus hν for the sample. The intersection of the straight line with the hν-axis determines the optical band gap energy Eg. The band gap of ZnO and Cu_2_O layers were found to be 3.2 and 2.0 eV corresponding. These values are suitable to the ideal band gap of the ZnO and Cu_2_O crystals (Lv et al. 2015).

### Electrical properties

**Fig. 5:**
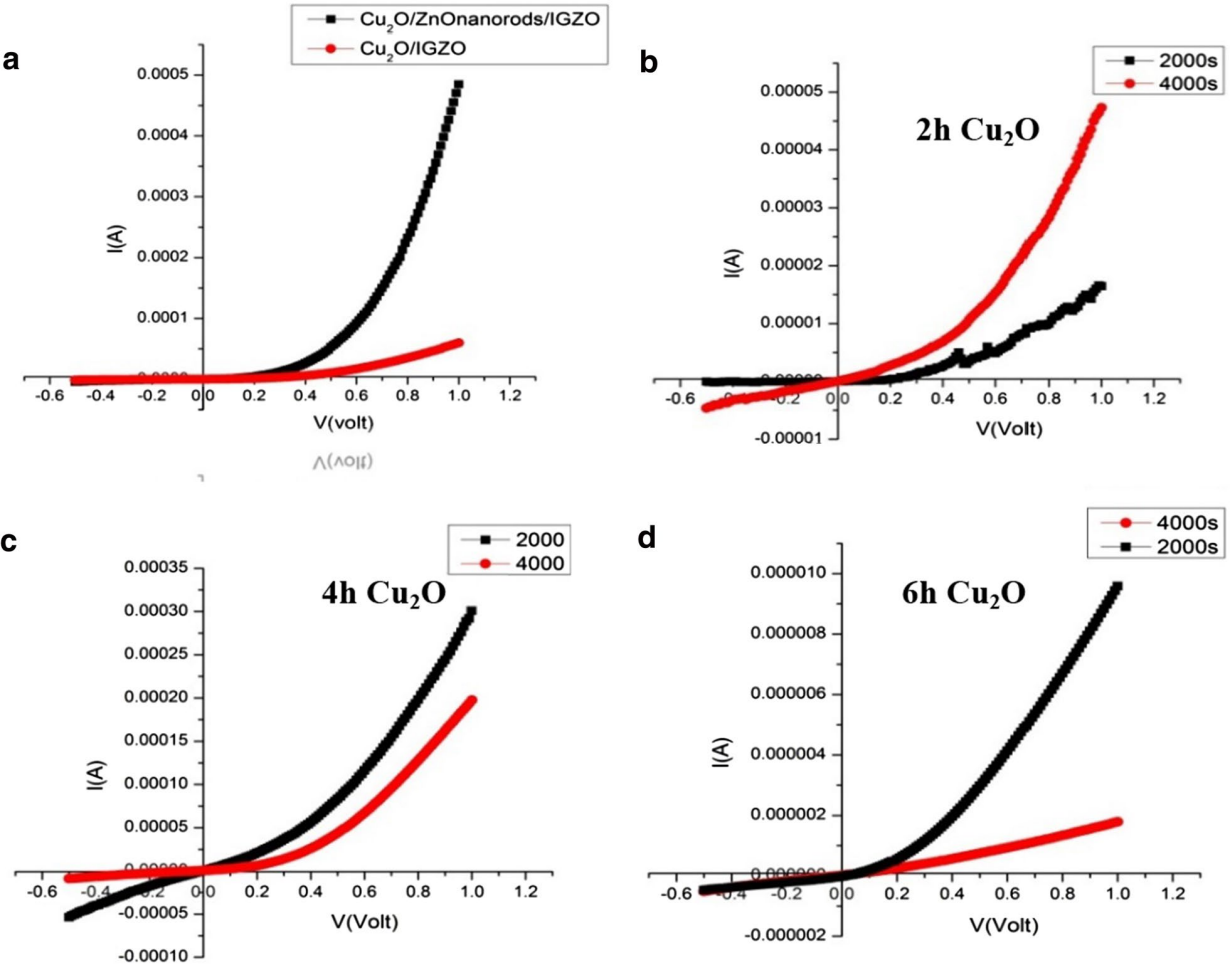
The dark I–V characteristic curves of: **a** Cu_2_O/IGZO and Cu_2_O/ZnO nanorods/IGZO structures. **b** Different growth-time of ZnO nanorod; 2000 and 4000 s with the same growth-time of Cu_2_O; 2 h. **c** Different growth-time of ZnO nanorod; 2000 and 4000 s with the same growth-time of Cu_2_O; 4 h. **d** Different growth-time of ZnO nanorod; 2000 and 4000 s with the same growth-time of Cu_2_O 6 h

Figure 5 presents the dark I–V characteristics of the p-Cu_2_O/i-ZnOnanorods/n-IGZO heterojunctions. The I–V characteristics of p–i–n heterojunctions exhibit a significant diode behavior. It indicates the fact that a potential barrier is formed in our heterojunction as Fig. 5a. With the forward bias, the carrier current passing barrier of the p-Cu_2_O/i-ZnO nanorods/n-IGZO structure is higher than the p-Cu_2_O/n-IGZO structure. Keeping stable deposition time of Cu_2_O layer in 2, 4, 6 h and changing the length of ZnO nanorods by increasing growth-time of rods from 2000 to 4000 s is presented in Fig. 5b–d. In the case of thinner Cu_2_O layers as Figs. 2c and 5b, the heterojunction with long ZnO nanorods get higher conductivity in forward bias. This is attributed to the contribution of large surface area of ZnO nanorods at interface which effectively increases the carriers transport capability (Chen et al. 2015). However, the leakage current is also increased in the reverse bias indicated the fact that many defects in ZnO nanorods such as vacancy, interstices and defects on the surface have worked as recombination centers (Perng et al. 2013). In the case of thicker Cu_2_O layers as Figs. 2e and 5c, d, the i-ZnO nanorods layer plays an important role as buffer layer between IGZO and Cu_2_O. In this case, the threshold voltage increases with increasing i-ZnO layer thickness that may be attributed to the increasing of potential barrier caused by balance of Fermi level in high crystalline ZnO, Cu_2_O structures. Moreover, the forward currents decreases because electrons meet more resistance when they pass through i-ZnO thicker layer.

**Fig. 6:**
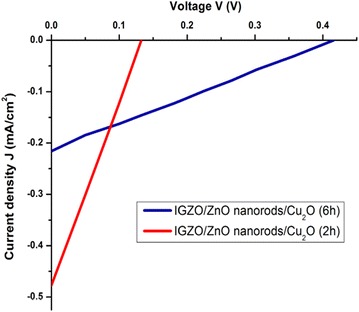
The J–V characteristic curves of Cu_2_O/ZnO-nanorods/IGZO structures in situations of different growth-time of Cu_2_O; 2 and 6 h with the same growth-time of ZnO nanorods; 2000 s

The carriers transport mechanisms and performance of the junction were deeply considered by the illuminated J–V characteristic curves shown on Fig. 6. Both structures of the p-Cu_2_O/i-ZnO nanorods/n-IGZO heterojunction were prepared with same deposition time of ZnO nanorods at 2000 s and changing deposition time of Cu_2_O layers for 2 and 6 h. Clearly, increasing in thickness of Cu_2_O lead to the increasing of open circuit-voltage from 0.14 to 0.41 V and decreasing of short circuit-current density from 0.47 to 0.21 mA/cm^2^. This indicated that the effecting of large surface area and one-direction conductivity of ZnO nanorods is only meaningful to thinner Cu_2_O layers (Fig. 2c). In this case, pair of hole-electron generated from absorption layer will be separated quickly to the contacts. However, the thinner Cu_2_O layer (Fig. 2c) effects to optical absorption capability and diffusion length of hole in IGZO layer, electron in Cu_2_O layer. Therefore, the current density is still low for both situations. Another reason is that the thinner Cu_2_O layer (Fig. 2c) leads to weak crystallinity of Cu_2_O and then reduces barrier potential at interface (Lv et al. 2013). This is observed obviously with the increasing of open circuit-voltage via increasing the depth of Cu_2_O layer. Generally, ZnO nanorods layers have contributed to carriers-collection and carriers-separation capability of heterojunction. Adjusting the length of ZnO nanorods, the thickness of Cu_2_O combined with improving defects in rods and on surface of rods are necessary to improve conversion efficiency of the p-Cu_2_O/i-ZnO nanorods/n-IGZO heterojunction.

## Conclusion

In this work, the p-Cu_2_O/i-ZnO nanorods/n-IGZO heterojunction was fabricated by electrochemical, sputtering method. The ZnO nanorods layer was deposited between two layers: n-IGZO and p-Cu_2_O to investigate the carrier transport mechanisms and performance of the junction. The clear boundaries were observed between two layers. The absorption range of the IGZO/ZnO nanorods/Cu_2_O heterojunction was expanded from 400 to 800 nm because of the high absorption coefficient in the visible range of the Cu_2_O layer. It is found that the length ZnO nanorods layer has contributed to carriers-collection and carriers-separation capability of heterojunction.
